# Metastasis of Disease from the Bowels to the Brain

**Published:** 1836

**Authors:** 


### Art. IV.—Of Metastasis of disease from the Bowels to the Brain, occasioned by Opium.

Sometime in February last, we were called to visit a child sixteen months old, which appeared te be laboring under some intestinal irritation, but before we arrived another physician had prescribed, and we departed after examining the symptoms of the disease, which we did before we were aware that the child had taken any medicine. On the night following our first visit, we were again summoned to the same patient, the first physician refusing to attend, when we found it in a state of deep coma, in which it had been for seventeen hours, apparently the result of a narcotic. We examined the medicine which had been left, and found that it contained opium, hence we concluded that too much had been administered, by mistake or otherwise, and proceeded to prescribe accordingly.—We ordered an emetic, followed by strong cathartics, with cold applications to the head, and departed. On the following morning we found that the medicine had operated well, but the coma with a slow full pulse, and little heat of the skin still remained. The pupils of the eyes presented no unnatural appearance; there was no stertor, and but little difficulty of breathing; the skin was soft, and apparently healthy, and the evacuations natural in appearance. Although the child took the breast, it could not be aroused even by the loudest noises. We ordered the warm bath, with cold water to the head, and hydrogogue cathartics. At noon the medicine had operated, and the excretions appeared healthy, but the coma still remained, and the child appeared to be in a sweet sleep. We now used the cold dash with no effect. At night we directed blisters to the head, and supperior portion of the spinal column, with repeated cathartics, in order to keep up the discharge from the bowels, as they were extremely torpid, except when under the influence of drastic cathartics. The blisters drew well, but the coma continued. Still the child was sensible to external impressions, for upon picking the skin in any part of the body, it would immediately give evidence of feeling. It had been now three days in the situation in which we found it, with but little variation of symptoms, and no prospect of relief. We resorted to venesection, but could get no blood, except from the veins of the neck. This, however, was attended with no beneficial result. Blisters were now extensively used over the abdomen, with terebinthinate cathartics and irritating frictions to the extremities and to the spinal column, but the patient appeared still to sleep quietly. Here we requested a consultation, and Dr. Ridgey was called to our assistance.—He observed a little enlargement of the pupil of the right eye, which escaped us, but the symptoms otherwise continued the same. Sensation presented no lesion in any external part of the body. The former course of treatment was persevered in, with occasional modifications, for thirteen days, when the child died. We could not get permission to examine the body.

Several other physicians, besides the one called in consultation, saw this patient, but did not suggest any alteration in the treatment.

This case presents a singular transfer of disease, from the abdominal vicera to the brain, which no irritation on the mucous tissue could restore. When we first saw the patient, it had no symptoms of cerebral disease whatever, but on the contrary, the bowels alone were affected. They had been deranged for several weeks, before any physician was called, and it is probable that the one who first saw it, thought the disease only the result of a simple irritation, which a narcotic would at once relieve. As the discharges, however, indicated much lasion, in the hepatic vicera, mercurials should by all means have preceded or accompanied the opiates. Mercurials in our hands readily procured billious evacuations, but the irritation could not be removed from the brain.

It is worthy of remark, that we had for a long time attended on this family, and always found that opium, in any form, produced unpleasant symptoms in this child, consequently, we had always forbiden its use. This idiosyncracy may have contributed much to the results of its use, in this case, for we cannot believe that a very great quantity had been administered. The physician left two portions, with directions that if the first failed to compose the child, in the course of a few hours, the second was to be given; but it threw the patient into a sleep in the course of two hours, from which it never awoke, although it appeared to remain easy, with no other symptom of cerebral irritation, for thirteen days. W. W.

